# Targeting Energy Metabolism in *Mycobacterium tuberculosis*, a New Paradigm in Antimycobacterial Drug Discovery

**DOI:** 10.1128/mBio.00272-17

**Published:** 2017-04-11

**Authors:** Dirk Bald, Cristina Villellas, Ping Lu, Anil Koul

**Affiliations:** a Department of Molecular Cell Biology, AIMMS, Faculty of Earth and Life Sciences, Vrije Universiteit Amsterdam, Amsterdam, the Netherlands; b Infectious Diseases and Vaccines Therapeutic Area, Janssen Research & Development, Beerse, Belgium; Harvard School of Public Health; Harvard Medical School

**Keywords:** antibiotics, *Mycobacterium tuberculosis*, energy metabolism, oxidative phosphorylation

## Abstract

Drug-resistant mycobacterial infections are a serious global health challenge, leading to high mortality and socioeconomic burdens in developing countries worldwide. New innovative approaches, from identification of new targets to discovery of novel chemical scaffolds, are urgently needed. Recently, energy metabolism in mycobacteria, in particular the oxidative phosphorylation pathway, has emerged as an object of intense microbiological investigation and as a novel target pathway in drug discovery. New classes of antibacterials interfering with elements of the oxidative phosphorylation pathway are highly active in combating dormant or latent mycobacterial infections, with a promise of shortening tuberculosis chemotherapy. The regulatory approval of the ATP synthase inhibitor bedaquiline and the discovery of Q203, a candidate drug targeting the cytochrome *bc*_1_ complex, have highlighted the central importance of this new target pathway. In this review, we discuss key features and potential applications of inhibiting energy metabolism in our quest for discovering potent novel and sterilizing drug combinations for combating tuberculosis. We believe that the combination of drugs targeting elements of the oxidative phosphorylation pathway can lead to a completely new regimen for drug-susceptible and multidrug-resistant tuberculosis.

## INTRODUCTION

Tuberculosis (TB) along with HIV ranks as a leading cause of death worldwide. In 2015, tuberculosis killed 1.4 million people and 10.4 million people are estimated to have fallen ill with TB (1). TB mortality has fallen since 1990; however, the rise of multidrug-resistant (MDR) and extremely drug-resistant (XDR) strains of *Mycobacterium tuberculosis* represents a serious health challenge. Whereas drug-sensitive TB can be treated by 6 months of chemotherapy with the current four-drug frontline regimen, to cure MDR-TB at least 18 to 24 months of therapy with four to six drugs, including a fluoroquinolone and one injectable agent, is required (2, 3). MDR-TB is defined as resistance to at least two of the four current frontline antibacterials; XDR strains of *M. tuberculosis* additionally are resistant to fluoroquinolones and at least one second-line drug (1). About 3% of new cases and 20% of treated tuberculosis patients are infected with MDR-TB; among these, about 9% are XDR cases (1).

The extended chemotherapy for TB and in particular for MDR-TB is a major factor for development of drug resistance (4). To achieve global control of this epidemic, there is an urgent need for new anti-TB drugs that can target MDR and XDR strains and shorten treatment duration for both drug-sensitive and drug-resistant TB (4–6). To address this unmet medical need, new anti-TB drugs have been discovered and new treatment regimens currently are being evaluated in (pre)clinical trials (5, 7). Two of these agents, the diarylquinoline bedaquiline (BDQ) and the nitroimidazo-oxazole delamanid, have received accelerated regulatory approval by the U.S. Food and Drug Administration (FDA) (7). The efficacy and safety of these drugs are currently being further evaluated in phase 3 clinical trials in order to define their role in TB chemotherapy regimens.

Bedaquiline (BDQ) has been identified as a potent inhibitor of mycobacterial ATP synthase (8–10), thereby validating oxidative phosphorylation as a target pathway for antibacterials. Within the last decade, several components of this central pathway have been identified as antibacterial targets, and small-molecule inhibitors of oxidative phosphorylation are active against drug-sensitive and drug-resistant TB. Therefore, presently there is strong interest in exploiting oxidative phosphorylation as a target for new antimycobacterial drugs and drug combinations. In this review, key concepts underlying the targeting of ATP synthesis in *Mycobacterium tuberculosis* are presented with the aim of developing new drugs and drug combinations active on diverse components of this central metabolic pathway.

### Oxidative phosphorylation in *Mycobacterium tuberculosis* as target of antibacterials.

**FIG 1:**
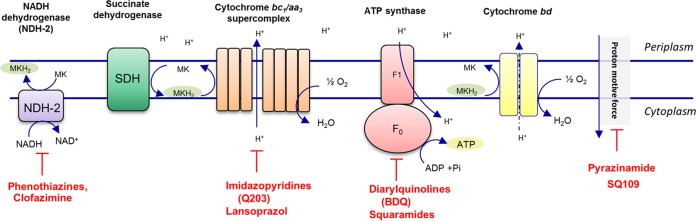
Oxidative phosphorylation in *M. tuberculosis*. Electrons derived from NADH are fed into the electron transport chain by NADH dehydrogenase, leading to the reduction of the menaquinone pool (MK/MKH_2_). In *M. tuberculosis,* the type I NADH dehydrogenase, the homologue of complex I in mitochondria, is dispensable for growth. Instead, mycobacteria employ the type II NADH dehydrogenase (NDH-2), which is present in two copies in *M. tuberculosis*. The menaquinone pool can also be reduced by alternative electron donors, e.g., via the succinate dehydrogenase (SDH). *M. tuberculosis* has two succinate dehydrogenase enzymes (Sdh-1 and Sdh-2) and one fumarate reductase, which catalyzes the reverse reaction. From the menaquinone pool, electrons can be transferred to the cytochrome *bc*_1_ complex. In mycobacteria, the cytochrome *bc*_1_ complex forms a supercomplex with the cytochrome *aa*_3_-type terminal oxidase, which transfers the electrons onto oxygen. Alternatively, oxygen can be reduced by a cytochrome *bd*-type terminal oxidase, which directly accepts electrons from the menaquinone pool. During electron transport along the respiratory chain, protons are pumped across the membrane, leading to a proton motive force (PMF). The energy of the PMF can be used by ATP synthase for synthesis of ATP.

Bacteria can produce ATP by substrate-level phosphorylation of fermentable carbon sources or by oxidative phosphorylation using the oxidative phosphorylation pathway. High-density mutagenesis and deletion mutant studies have revealed that *M. tuberculosis* and related mycobacterial strains apparently cannot gain enough energy by substrate-level phosphorylation and need oxidative phosphorylation for growth (11, 12). In oxidative phosphorylation, the protein complexes of the respiratory chain establish a proton motive force (PMF) across a biomembrane; subsequently, the energy of this PMF is used by ATP synthase for the production of ATP (13–16) (please see Fig. 1 for an overview). In *M. tuberculosis,* the inflow of electrons and maintenance of the proton motive force as well as the final step, synthesis of ATP, are essential for growth and survival (11, 12, 17). In line with this observed essentiality, these different functionalities of oxidative phosphorylation can be targeted by small-molecule inhibitors, which can prevent respiratory electron transport, break down the proton motive force, or block the production of ATP. An overview of small-molecule inhibitors targeting oxidative phosphorylation components and their current state of (pre)clinical development is presented in Table 1.

**TABLE 1:** Drugs targeting oxidative phosphorylation and their current state of clinical development

Drug class (lead compound)	Target	Screening/discovery	Phase of clinical development	Clinical use	Reference(s)
Clofazimine	NDH-2	Antileprosy drug repurposed for tuberculosis	Approved (leprosy)	MDR-TB	20, 74
Phenothiazines (thioridazine)	NDH-2	Neuroleptic compound repurposed for tuberculosis	Approved	MDR-TB	18, 19, 21
Imidazopyridines (Q203)	Cytochrome *bc*_1_ complex	Phenotypic screening using *M. tuberculosis* in infected macrophages	Phase 1		23
Diarylquinolines (BDQ)	ATP synthase	Phenotypic screening using *M. smegmatis*	Approved (phase 3)	MDR-TB	8, 9, 53
Squaramides	ATP synthase	Screening for ATP synthesis inhibition in *M. smegmatis* membranes	Preclinical		26
Pyrazinamide	Proton motive force	Nicotinamide analog tested directly in murine model	Approved	DS^a^ and MDR-TB	29, 31
SQ109	Proton motive force	Phenotypic screening using *M. tuberculosis* (library of 1,2-ethylenediamine compounds)	Phase 2	DS and MDR-TB	30, 77

a DS, drug sensitive, refers to *M. tuberculosis* isolates with no resistance to first-line antibiotics.

The phenothiazine drugs as well as clofazimine target the type II NADH dehydrogenase (NDH-2), the point of entry of electrons into the respiratory chain (Fig. 1) (18–20). Clofazimine is approved for treatment of leprosy (Table 1) and currently is being repurposed as anti-TB drug. Thioridazine and other phenothiazines are approved antipsychotic drugs whose applicability to the treatment of TB currently is under consideration (21). The cytochrome *bc*_1_ complex has been validated as a target of the imidazopyridines and related compounds (22–26). Imidazopyridine lead compound Q203, discovered by a phenotypic screen against *M. tuberculosis* in macrophages and subsequent chemical optimization (23), currently is in phase 1 clinical trials. The cytochrome *bc*_1_ complex was also reported as a target of the gastric proton pump inhibitor lansoprazole, identified in a host-cell-based screen of approved pharmacophores on *M. tuberculosis* (27). Small molecules of the diarylquinoline class, discovered by a phenotypic high-throughput screen on *Mycobacterium smegmatis*, inhibit the ATP synthase of *M. tuberculosis* and related mycobacterial strains (8–10, 28). Diarylquinoline lead compound BDQ has been approved by the U.S. Food and Drug Administration (FDA) and the European Medicines Agency (EMA) for treatment of multidrug-resistant tuberculosis. A recent high-throughput screen for inhibition of ATP synthesis in mycobacterial membrane vesicles and subsequent biochemical deconvolution identified the squaramides as a new class of ATP synthase inhibitors (26).

Several antibiotics and candidate drugs can collapse the proton motive force in mycobacteria, including pyrazinamide (29), which is part of the current frontline anti-TB regimen, and analogs of the diamine-based drug SQ109 (30). Both pyrazinamide and SQ109 (analogs) likely have multiple cellular targets (30, 31), and it is unknown to what extent inhibition of bioenergetic functions contributes to the antimycobacterial activity of these drugs.

### Toward expanding oxidative phosphorylation as target space.

Hitherto, high-throughput screening efforts have mainly yielded classes of small-molecule inhibitors active on either ATP synthase (8, 26) or the cytochrome *bc*_1_ complex (23, 25, 26). The reasons for this preference are obscure but may be related to the central role of these two enzymes in either establishing or utilizing the proton motive force. The cytochrome *bc*_1_ complex also displays two substrate (quinone/quinol) binding sites that may be able to accommodate a variety of small-molecule inhibitors.

Although highly active drugs are available against the currently validated targets in oxidative phosphorylation, it is desirable to further extend this target space. As an example of a currently not exploited target in oxidative phosphorylation, the succinate dehydrogenase Sdh-1 has been pinpointed as a key regulator of mycobacterial oxidative phosphorylation during adaptation to low-oxygen environments (32), whereas fumarate reductase, which catalyzes the reverse reaction, was found instrumental in maintaining the proton motive force under anaerobic conditions (33). Screening using focused biochemical assays covering only part of the oxidative phosphorylation pathway (26) and/or phenotypic screening under hypoxia may be applied for discovery of new hits targeting these metabolic regulator enzymes. The cytochrome *bd*, an alternative terminal oxidase of the mycobacterial respiratory chain (Fig. 1), represents another example of a largely unexplored target. Cytochrome *bd* is important for growth under low oxygen tensions as well as for survival in the mammalian host (34–36). Upon mutation or chemical inhibition of the cytochrome *bc*_1_ complex, several *M. tuberculosis* strains are able reroute respiratory electron flux to cytochrome *bd* (24, 37, 38), leading to decreased sensitivity for cytochrome *bc*_1_ inhibitors (24, 25). Cytochrome *bd* can also protect mycobacteria against bedaquiline (39–42) and clofazimine (41). Targeting cytochrome *bd* may therefore be an effective strategy for weakening the mycobacterial response to antibacterials. In this regard, the recently reported crystal structure of cytochrome *bd* (43) may assist in the design of small-molecule inhibitors.

Interestingly, a recent study suggested that lipophilic, cationic tuberculosis drugs from a broad spectrum of chemical classes can dissipate the proton motive force in *M. smegmatis* (44). The significance of this finding and the potential contribution of uncoupling to the drugs’ bactericidal activity against *M. tuberculosis* need to be further examined. It may turn out that more drugs than previously thought exert their bactericidal activity in part via interfering with the proton motive force. This would represent a significant and unexpected extension of the oxidative phosphorylation target space.

### Selective inhibition of oxidative phosphorylation.

Avoiding toxicity is an important challenge for development of any drug. In particular, drugs active on bacterial pathways that are also present in eukaryotic systems may inhibit the eukaryotic homologue, thereby triggering target-based toxicity. As the oxidative phosphorylation pathway is largely conserved between prokaryotes and eukaryotes, this pathway has long been regarded as an unsuitable target of antibacterials. Whereas the general toxicity of a drug is difficult to predict, antibacterials have been discovered that selectively inhibit oxidative phosphorylation in mycobacteria compared with the homologous pathway in the human host, thereby minimizing or eliminating target-based toxicity.

The imidazopyridine lead compound Q203 caused pronounced inhibition of mycobacterial growth at nanomolar concentrations without significant impact on human cells in culture (23). In line with this observation, Q203 strongly suppressed ATP synthesis by mycobacterial membrane vesicles but did not influence respiration in human cell lines (37). Here, high-resolution structural data of the mycobacterial cytochrome *bc*_1_ complex with bound drugs are necessary for a better understanding of the molecular factors underlying drug selectivity.

Similarly, mycobacterial ATP synthase displays high affinity for the ATP synthase inhibitor BDQ compared with the human homologue (45). Recently, a high-resolution structure of subunit c of mycobacterial ATP synthase showed that BDQ was bound to its target in a lock-and-key fashion (46). The surface profiles and binding site geometries were virtually identical in all mycobacteria, whereas in other prokaryotic and eukaryotic c-rings the observed drug-target interaction would be sterically hindered (46). As observed for BDQ, squaramide lead compound 31f also displayed selective inhibition of mycobacterial ATP synthesis confirmed by counterscreening of hits against mammalian ATP synthesis (selectivity index, >700) (27). Based on resistance mutations and docking studies, a binding pocket for this drug was postulated at the interface between subunit a and subunit c of mycobacterial ATP synthase (26); however, this mode of binding needs to be confirmed by X-ray crystallography.

### Inhibitors of oxidative phosphorylation are potent under *in vivo* conditions.

In the lungs of the mammalian host, *M. tuberculosis* encounters unfavorable environmental conditions, including nutrient limitation, low oxygen tensions, or low/high pH values (47). These conditions can trigger extensive adaptations in mycobacterial metabolism: the bacteria can enter a dormant state with very slow or no growth (48), and they can use host-derived fatty acids instead of carbohydrates as an energy source (49). The need for elimination of dormant bacteria persisting in the host is a major determinant for the extended chemotherapy required for combating both acute and chronic or latent tuberculosis infections (4).

In the dormant metabolic state, *M. tuberculosis* maintains 5- to 10-fold-lower cellular ATP levels than do replicating bacteria (17, 50). Due to its low cellular energy levels, dormant *M. tuberculosis* does not carry out many biosynthetic activities, a major factor for its low susceptibility to antibacterials targeting protein, DNA, or cell wall biosynthesis (51). In contrast, a drug-induced decrease of already low energy levels in dormant mycobacteria may make these bacilli susceptible to inhibitors of oxidative phosphorylation. Exemplifying this concept, the bactericidal activity of BDQ against dormant bacteria *in vitro* was found to be higher than its activity against replicating *M. tuberculosis* (17, 50), and killing by BDQ was correlated with an additional, pronounced drop of cellular ATP (48). Similarly, Q203 efficiently decreased ATP levels in *M. tuberculosis* under anaerobic conditions (23). In line with its strong *in vitro* activity against dormant *M. tuberculosis*, BDQ revealed sterilizing activity in mouse tuberculosis infection models (52) and caused accelerated culture conversion in tuberculosis patients (53).

Interestingly, the frontline drug pyrazinamide, which is regarded as the component of the current anti-TB regimen with the highest activity on dormant *M. tuberculosis*, has mechanistically also been linked to energy metabolism (29). Pyrazinamide is a prodrug whose active entity, pyrazinoic acid, can interfere with the proton motive force (29). However, the mechanism of action of pyrazinamide appears to be pleiotropic, and it is unclear to what extent inhibition of energy metabolic functions contributes to the drug’s sterilizing activity.

In addition to downregulating biosynthetic pathways, mycobacteria can also adapt their metabolism to the host environment by employing alternative energy and carbon sources. *M. tuberculosis* residing in human macrophages mainly uses host-derived cholesterol and fatty acids as an energy supply instead of fermentable energy sources (48). Mycobacteria located in macrophages represent a therapeutically particularly recalcitrant subpopulation of *M. tuberculosis* in the human host, against which the activity of most frontline anti-TB drugs is significantly reduced (51). In contrast, for Q203 the MIC for growth inhibition (MIC_50_) was found to be ~10-fold lower for *M. tuberculosis* in a human macrophage model than for the bacteria grown in broth culture (23). Likewise, BDQ displayed strong activity against *M. tuberculosis* in macrophages, exceeding the drug’s activity against extracellular mycobacteria (54). It can be speculated that this enhanced potency of Q203 and BDQ against *M. tuberculosis* in human macrophages is, at least in part, due to the metabolic dependency of these bacteria on fatty acids as an energy source. Whereas fermentable energy sources such as glycerol or glucose allow for ATP synthesis by glycolysis or by oxidative phosphorylation, with fatty acids as an energy source the bacteria cannot utilize the glycolytic pathway, and ATP production is restricted to oxidative phosphorylation. Consistent with this notion, *M. tuberculosis* grown *in vitro* on fatty acids displayed enhanced susceptibility for bedaquiline compared with standard growth medium containing glycerol or glucose (39). It needs to be evaluated if increased activity against bacteria growing *in vitro* on fatty acids and against *M. tuberculosis* in macrophages is shared by other small-molecule inhibitors targeting the oxidative phosphorylation pathway.

Downregulation of biosynthetic functions in *M. tuberculosis* and rerouting of central metabolism toward utilization of nonfermentable constitute are two key aspects of metabolic changes of mycobacteria residing in the human host and represent a major challenge for existing and future anti-TB chemotherapy. Inhibitors of oxidative phosphorylation appear particularly suitable for combating these metabolically adapted bacteria.

### Depletion of bacterial energy reserves may inactivate efflux pumps.

Inhibitors of bacterial energy metabolism may prove highly suitable in drug combination regimens due to interference with drug efflux. The contribution of efflux pumps to resistance in *Mycobacterium tuberculosis* has long been underestimated as drug resistance in many clinical isolates is due to the acquisition of mutations in genes encoding drug targets. In recent years, it became evident that efflux pumps can be a determinant for antibacterial sensitivity in *M. tuberculosis* (55–60). *M. tuberculosis* located in human macrophages and macrophage-like cell lines displayed prominent efflux pump activity, mediating resistance to a variety of antitubercular drugs, including rifampin, moxifloxacin, pretomanid, linezolid, and bedaquiline (55, 57). Inhibition of efflux therefore is highly desirable and intensely pursued in the pharmaceutical industry. Current approaches include optimization of small-molecule compounds active on efflux pumps (61) or structurally redesigning existing drugs in order to evade expulsion by efflux pumps (62).

**FIG 2:**
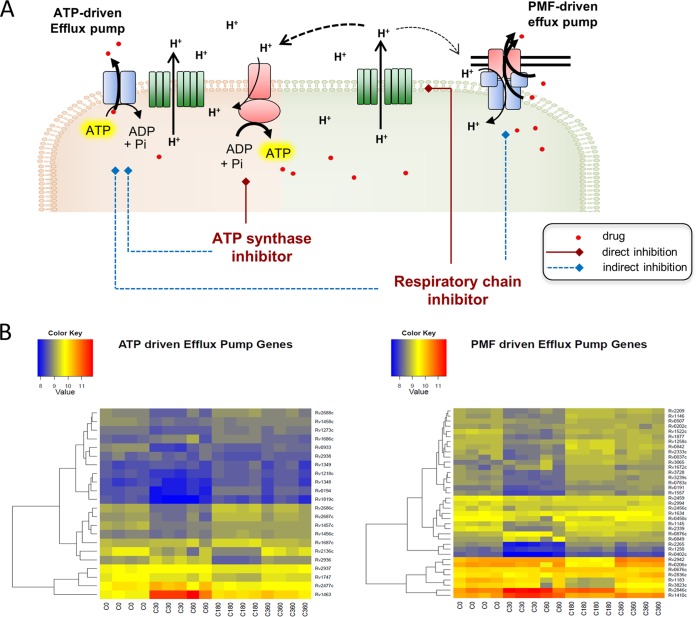
Effect of inhibitors of oxidative phosphorylation on function and expression of efflux pumps. (A) Inhibitors of oxidative phosphorylation can act at 2 different levels: limiting the electron flow through the respiratory chain or inhibiting directly the production of ATP. Inhibitors of the ATP synthase (red lines, direct inhibition) cause the ATP pool to drop, thus decreasing the function of primary transporters (blue dashed lines, indirect inhibition), which use ATP directly to fuel transport through the membrane. The inhibitors of the electron flow in the respiratory chain lower the generation of proton motive force (PMF), which results in less function of secondary transporters (PMF driven) and in less ATP production, which affects the function of ATP-driven efflux pumps. As a result, efflux pumps cannot extrude their substrates as efficiently as in the absence of inhibitors of oxidative phosphorylation, and if used in combination with other drugs, the intracellular concentration of these drugs can be increased and their activity can be enhanced. Note that in mycobacteria ATP synthase synthesizes ATP from ADP and P_i_ and apparently does not invert its function (79). (B) Effect of bedaquiline on the transcription levels of efflux pumps in *M. tuberculosis*. RNA levels of ATP- and PMF-driven efflux pumps after 0, 30, 180, and 360 min of treatment with bedaquiline are shown. The RNA profile shows temporary downregulation for the majority of efflux pump genes after 30 min of exposure to bedaquiline. The color scales represent log_2_ fold changes in gene expression.

Draining the energy supply of efflux pumps may turn out to be an alternative strategy to impede drug efflux. All efflux pumps need energy to transport their substrates against the concentration gradient out of the bacterial cell. This energy can be supplied as ATP, used by the primary transporters, e.g., the ATP binding cassette (ABC) efflux pumps, or alternatively as proton motive force, which is utilized by the secondary transporters, e.g., the resistance nodulation cell division (RND)-type pumps. For drug extrusion, a sufficiently high proton motive force and ATP levels have to be maintained by the bacterial energy metabolism. Compounds blocking oxidative phosphorylation therefore may indirectly interfere with efflux pump function (14, 56, 63) (Fig. 2A).

Exemplifying this concept, it has been shown that treatment of *M. smegmatis* with chlorpromazine or thioridazine, which both are inhibitors of NDH-2, enhanced the intracellular levels of ethidium bromide (64). Similarly, the ATP synthase inhibitor BDQ interfered with ethidium bromide efflux in *M. smegmatis* (65). Efflux inactivation correlated with a sufficiently high decrease of cellular ATP levels, thereby providing a link between efflux inhibition and oxidative phosphorylation (65). This inactivation of efflux by an oxidative phosphorylation inhibitor may well lead to increased intracellular levels of companion drugs, thereby enhancing their potency (Fig. 2A).

In addition to blocking the energy supply of efflux pumps required for drug extrusion, oxidative phosphorylation inhibitors may also diminish the abundance of bacterial efflux pumps. The metabolic response to BDQ revealed decreased levels of the majority of efflux pump proteins and their mRNA transcripts in *M. tuberculosis* (39) (Fig. 2B). This downregulation, likely triggered by a reduced cellular energy charge, may further contribute to the diminished efflux of small-molecule drugs. The signaling transduction routes linking efflux pump expression to the cellular energy charge need to be elucidated.

The observed ability to inactivate and/or downregulate efflux pumps may in part explain why the emergence of resistance to frontline antituberculosis drugs was lower in the presence of bedaquiline (53). The ability to deplete bacterial energy reserves, thereby interfering with drug efflux, may make inhibitors of bioenergetic pathways ideally suitable for the combination regimens needed to tackle multidrug-resistant *Mycobacterium tuberculosis*.

### Delayed kill by energy metabolism inhibitors: rethinking early clinical characterization of antituberculosis drug candidates.

Compounds interfering with oxidative phosphorylation display pronounced bactericidal activity against *M. tuberculosis in vitro* and *in vivo* (39, 52) and may therefore make a significant contribution to shortening tuberculosis chemotherapy. However, promising new compounds with this mechanism of action can be underestimated by currently used protocols for the characterization of drug candidates, as their bactericidal activity within the first days of treatment generally is minimal (23, 39, 52, 66). The bactericidal activity in the initial phase of treatment (early bactericidal activity [eBA]) constitutes an important parameter for the evaluation of new drug candidates. eBA studies quantify *Mycobacterium tuberculosis* in sputum from pulmonary tuberculosis patients and measure the ability of antituberculosis treatments to reduce the mycobacterial burden in patients during the first days of therapy.

This discrepancy between potent long-term activity and low initial activity has been observed for Q203 in a mouse model of tuberculosis, where the reduction in bacterial counts was less than 1 order of magnitude in the first 2 weeks of treatment but more than 2 orders of magnitude in the following 2 weeks (23). BDQ displayed low initial bactericidal activity (≤1-log_10_-unit kill) within the first week of treatment in early bactericidal activity studies with human tuberculosis patients (66, 67) and against *M. tuberculosis in vitro* (39, 54). Similarly, clofazimine lacked bactericidal activity within the first week of treatment in a mouse model of tuberculosis (68). Interestingly, the lack of eBA within the first days of treatment could not be overcome by increasing the dosage of the drugs (23, 39, 66, 68). Consequently, drug assessment based on eBA may underestimate the potency of inhibitors that display a delayed onset of bacterial killing.

A variety of factors may contribute to the delay in kill, such as drug binding to host plasma proteins and lack of drug penetration into granulomas where the bacteria can reside, as well as other pharmacokinetic factors. However, as three different inhibitors of the oxidative phosphorylation pathway are all slow acting, the reasons for the delay are likely due to microbiological and molecular factors underlying the delayed onset. Cellular energy reserves need to be depleted to a sufficient degree in order to interfere with bacterial viability. BDQ treatment of *M. tuberculosis* triggered metabolic remodeling to counteract cellular ATP depletion that enables maintenance of ATP levels sufficient for bacterial viability during the initial days of treatment (39). As an alternative mechanism of (delayed) killing by BDQ, futile proton cycling across the mycobacterial cytoplasmic membrane has been proposed, which in a time-dependent manner leads to collapse of the proton motive force (42). It can be expected that the delay in bacterial killing observed for other oxidative phosphorylation inhibitors such as Q203 and clofazimine is due to a similar metabolic remodeling, which transiently preserves bacterial energy reserves. Therefore, eBA studies with this type of drug need to be evaluated with caution. eBA studies with a time frame up to 14 days (“extended eBA studies”), which recently have been carried out for characterization of several new tuberculosis drug combination regimens, e.g., for the combination of SQ109 with rifampin (69), seem advisable for characterization of new oxidative phosphorylation inhibitors alone or in combination regimens.

### Inhibitors of oxidative phosphorylation as key components of new anti-TB regimens.

Small-molecule inhibitors targeting oxidative phosphorylation are highly active against multidrug-resistant and extensively drug-resistant tuberculosis (70, 71). However, as *Mycobacterium tuberculosis* can quickly develop resistance to individual drugs, the development of efficient combination regimens is crucial for shortening anti-TB treatment duration (72). Intriguingly, several inhibitors of oxidative phosphorylation perform very efficiently in combination with first- and second-line drugs as well as experimental anti-TB drugs.

The NDH-2 inhibitors thioridazine and chlorpromazine acted synergistically with first- and second-line drugs such as isoniazid, rifampin, and amikacin on MDR-TB clinical isolates *in vitro* (58). These drugs also potentiated the activity of frontline drugs against drug-sensitive and drug-resistant *M. tuberculosis* strains in an MGIT model (73). A second NDH-2 effector, clofazimine, enhanced the activity of a second-line regimen against an isoniazid-resistant strain of *M. tuberculosis* in a mouse model of infection (74). Clofazimine-containing drug combinations also significantly reduced the time for lung culture conversion in a mouse model of drug-susceptible TB (75).

For the ATP synthase inhibitor BDQ, highly potent combination regimens against *M. tuberculosis* in mouse models have been reported in combination with the frontline drug pyrazinamide (76) and with the third-line drug clofazimine (52). In human MDR-TB patients, addition of BDQ to the standard regimen in a phase 2 clinical trial reduced the conversion time to sputum-negative culture compared with placebo (53) and the combination of BDQ, pyrazinamide, and the nitroimidazole pretomanid showed high potency in a 14-day bactericidal activity study with pulmonary TB patients (71). BDQ also displayed synergy *in vitro* when applied together with the investigational compound SQ109 (77) as well as with piperazine-containing benzothiazinones (78).

The observed synergy may be due to various factors. Inactivation of efflux pumps, as detailed above, may contribute to the synergy. However, other factors such as killing of specific bacterial subpopulations, e.g., bacteria in a dormant metabolic state or residing in macrophages, likely contribute as well. It needs to be evaluated if synergy in drug combination regimens constitutes a general feature of antibacterials targeting oxidative phosphorylation. If yes, then utilizing combinations of two or more oxidative phosphorylation inhibitors may lead to potentiation of the activity of each individual drug. Illustrating this principle, recently it was demonstrated that the triple combination of BDQ, Q203, and clofazimine highly efficiently killed *M. tuberculosis in vitro* and in a macrophage model (37). The combination of oxidative phosphorylation inhibitors may allow for the design of completely new anti-TB regimens, independently of the presently used frontline drugs. This strategy is hitherto largely unexplored but may provide exceptionally useful in order to achieve the overarching aim of shortening TB treatment.
